# Prevalent Parental Practice Toward Drug Storage and Disposal

**DOI:** 10.7759/cureus.60449

**Published:** 2024-05-16

**Authors:** Hussain A Al Ghadeer, Jawad S Alnajjar, Jalal K Aldandan, Alla A Bokhamseen, Ali M Al Dandan, Mohammed A Almarzoq, Hussain J Alnajjar, Ali H Albuti, Mohammed A Almuhaini, Maryam A Alsalman, Shifa A Al Sabah

**Affiliations:** 1 Pediatrics, Maternity and Children Hospital, Al-Ahsa, SAU; 2 College of Medicine, King Faisal University, Al-Ahsa, SAU; 3 Pediatrics, King Faisal University, Al-Ahsa, SAU; 4 Pharmacy, King Fahad Hospital of the University, Al-Ahsa, SAU; 5 Pharmacology, Almoosa Health Group, Al-Ahsa, SAU; 6 Nursing, Primary Health Care, Al-Ahsa Health Cluster, Al-Ahsa, SAU

**Keywords:** saudi arabia, storage, medication, home, disposal

## Abstract

Introduction

The environment, healthcare services, and public safety can all be directly impacted by improper drug storage and disposal practices. It is unknown whether parents store drugs at home in accordance with recommended storage guidelines, despite the fact that storage conditions are strictly regulated and monitored at every stage of the drug supply chain prior to drug dispensing. Therefore, it is crucial to dispose of medications properly and store them at home to avoid the consequences.

Aim

The purpose of this study was to evaluate the drug storage safety measures used by parents to prevent unintentional drug poisoning in children.

Methodology

A structured questionnaire was used to conduct a cross-sectional, interview-based study on home medication storage, attitudes, and disposal practices between October 2023 and January 2024. We recruited parents who visited primary healthcare centers or pediatric clinics using a convenience sampling technique.

Results

All of the 353 returned questionnaires were valid for data entry and analysis. The mean age of the parents was 35.1 ± 11.9 years old and more than half of them 229 (64.9%) have bachelor’s degrees. The majority of drugs (271, 88.6%) were stored in the fridge, followed by bedrooms (26.8%). The medication classes that were stored the most frequently were analgesics (92.2%) and antihistamines (62.1%). The majority of parents (214, 69.9%) kept medications above adult eye level, even though only 28% did not keep them in safe and secure locations like locked drawers or boxes. Eighty percent (80%) disposed of unwanted medicines by throwing them in the trash, and only 10 (2.8%) returned them to the pharmacy.

Conclusions

Drug storage at home encourages self-medication, which has a number of negative effects. Over time, there has been an increase in the use of medications due to a rise in people's health-seeking awareness and behavior on a global scale. Therefore, this study may be used as a guide by national policy-makers for pharmaceutical disposal and storage management. Moreover, it might help in raising public awareness of the importance of pharmacists in the society and the safe handling and storage of medications at home.

## Introduction

Medications are produced and utilized in enormous quantities, and their use and diversity are expanding yearly. The use of medications is lifesaving, but improper consumption can be catastrophic. Thus, unsafe storage practices could expose people to danger, especially children [1-3]. Appropriate conditions of security, humidity, ventilation, temperature, and light should be ensured. All medicinal products must be stored under the manufacturer’s directions and within the terms of product authorizations [4]. Based on prior studies, children younger than six years are at a higher risk of medication poisoning due to their curiosity, inability to read the labels, and their biological vulnerability to poisoning. Despite their curiosity, children are more susceptible to unintentional poisoning when their parents store medications in an unsafe manner. Another study concurred that the main risk factors were a lack of adult supervision and free access to drugs [5-7]. As a result, thousands of children aged six and younger visit the emergency department due to unsupervised consumption of oral medications [8]. A tertiary care teaching hospital in Riyadh City showed that medication poisoning accounted for nearly half (12/44, 47.7%) of the reported poisoning cases in children less than six years [9]. Enormous storage of medication can be attributed to several factors, including excessive physician prescription, excessive purchasing, and nonadherence to treatment. In addition, there was a significant relationship between the number of medications stored in households and factors such as insurance coverage, chronic illnesses (such as diabetes mellitus, hypertension, cardiovascular disease, and cancer), siblings not working in health-related jobs, higher economic status, and literacy of the father and parents who have children less than six years old [10-13].

Besides the poisoning risk, pharmaceutical products end up in the environment due to the public's incorrect disposal of medications, which accentuates the importance of understanding the disposal practices of medications among the populace [14,15]. It is critically important and a top priority to develop uniform guidelines for medication disposal and to regulate drug donations. Instructions for safe disposal should be routinely provided to patients by all healthcare professionals. Regardless of the type of medication, the most common method of medication disposal is through household trash, followed by flushing them down the toilet or sink [14]. 

Despite improvements in lifestyle, childhood poisoning remains a major source of illness and potentially even mortality in the capital city of Saudi Arabia, and this is due to a lack of knowledge [9,11]. Furthermore, it has an economic concern since families in Saudi Arabia and other Gulf nations paid a combined USD 150 million on medications that were never used [16]. In contrast to focusing on information, no previous studies have examined drug storage and disposal practices and attitudes. Therefore, this study aimed to assess drug storage and disposal practices and attitudes among parents in the Eastern region of Saudi Arabia.

## Materials and methods

Study design and selection criteria

This cross-sectional design study was conducted between October 2023 and January 2024 at King Faisal University, Al-Ahsa, Eastern Province of Saudi Arabia. The study aimed to assess the practice and attitude toward storage and disposal of drugs. It is based on a validated questionnaire tool that is administered among Saudi Arabian parents who have children less than six years old and live in the Eastern region. However, parents of a child of more than six years child, living outside the Eastern region, and arguing to participate were excluded. We used a convenience sampling method to recruit participants.

Data collection

A pilot study was done to assess its validity by distributing the Arabic version of the questionnaire among 30 participants after their agreement, and they were excluded later on from the final analysis. The questionnaire revolved around three sections. The first part is about demographic data and the agreement of participants to fill in questions, gender, age, educational level, having a child less than six years old, living in the Eastern region, and number of family members in the home. The second part is about storing medicines, whether there is a family member sufferer from chronic disease, if storing or not, the reason beyond storing medicines, number and types of stored medicines, where they are stored, and if there are instructions heard about storing medicines and from whom. The third section is about the disposal of medicines, the way of disposal, what improper disposal leads to, and instructions heard about disposal and from whom.

Ethical consideration and statistical analysis

An informed consent was provided to the participants before conducting the study. All data collected were kept confidential and solely used for research purposes, adhering to ethical guidelines for research involving human subjects. This study was approved by the Ethics Committee of King Faisal University (ethical approval code KFU-REC-2023-SEP-ETHICS1193). Statistical analysis was conducted by using Statistical Package for the IBM SPSS Statistics for Windows, version 26.0 (released 2019, IBM Corp., Armonk, NY). Descriptive statistics (means, SDs, and percentages), chi-square, and Fisher's exact test were used to analyze the data from the questionnaire. A p-value below 0.05 was considered statistically significant.

## Results

A total of 353 parents were included. The parents' ages ranged from 18 to more than 45 years, with a mean age of 35.1 ± 11.9 years old, and 36% of the participants were between the ages of 25-34 and 35-44 years old. Two hundred (56.7%) participants were females. As for education, 229 (64.9%) were university graduates, 87 (24.6%) had high school education, and 28 (7.9%) had a post-graduate degree. Considering the family size, 248 (70.3%) had families of four to eight persons, 87 (24.6%) had families of less than four persons, and 18 (5.1%) had families of more than eight persons. A total of 120 (34%) had a family member suffering from chronic illness (Table 1).

**Table 1:** Sociodemographic characteristics of the study parents in the Eastern region of Saudi Arabia (n = 353)

Sociodemographics	Number	Percentage %
Age in years	Mean: 35.1 ± 11.9 years old	
18-24	31	8.8%
25-34	127	36.0%
35-44	127	36.0%
> 45	68	19.3%
Gender		
Male	153	43.3%
Female	200	56.7%
Educational level		
Below high school	9	2.5%
High school	87	24.6%
University graduate	229	64.9%
Post-graduate	28	7.9%
Family size		
<4	87	24.6%
4-8	248	70.3%
>8	18	5.1%
Does anyone in your house suffer from chronic illness?		
Yes	120	34.0%
No	233	66.0%

A total of 158 (44.8%) received advice regarding safe storage of medications (Table 2). The sources of the information were leaflets attached to medication packages (69, 43.7%), pharmacists (41, 25.9%), doctors (31, 19.6%), friends (10, 6.3%), and social media (7, 4.4%). A total of 312 (88.4%) reported throwing drugs in the trash as the best method for disposal of unused medications, followed by sharing them with friends or family (16, 4.5%), returning them to a pharmacy or a hospital for disposal (10, 2.8%), and flushing them in the toilet (7, 2.0%). As for the parents' awareness about improper storage of medication effects, 199 (56.4%) reported decreased efficacy of the drug, followed by increased possibility of side effects (77, 21.8%) and change in the appearance of the drug (49, 13.9%). On the other hand, 22 parents (6.2%) think that storage methods of medications do not affect the quality or efficacy of the medication. A total of 241 (68.3%) stated that the improper disposal of medications affects the environment and health.

**Table 2:** Study parents' awareness and perception about drug storage and disposal in the Eastern region of Saudi Arabia (n = 353)

Awareness and perception of drug storage	Number	Percentage %
Have you ever received any advice regarding safe storage of medications?		
Yes	158	44.8%
No	195	55.2%
If yes, who gave you the advice?		
Leaflet attached to medication’s package	69	43.7%
Pharmacist	41	25.9%
Doctor	31	19.6%
Friend or family	10	6.3%
Social media	7	4.4%
In your opinion, what is the best method for disposal of unused medications?		
Throw them in the garbage	312	88.4%
Share them with friends or family	16	4.5%
Return them to a pharmacy or a hospital for disposal	10	2.8%
Other	8	2.3%
Flush them in the toilet	7	2.0%
In your opinion, what will improper storage of medication cause?		
Decrease the efficacy of the drug	199	56.4%
Increase the possibility of side effects	77	21.8%
Change in the appearance of the drug	49	13.9%
Storing medications in any method doesn’t affect the quality or efficacy of the medication	22	6.2%
Other	6	1.7%
Could the improper disposal of medications affect the environment and health?		
Yes	241	68.3%
No	19	5.4%
Don't know	93	26.3%

A total of 306 parents (86.7%) store medications at their house (Table 3). One hundred fifty-one (151, 49.3%) of them store one to five medications, and 63 (20.6%) store six to 10 medications. The most reported reasons for storing medications at the house were possible future use (205, 67%), daily use (187, 61.1%), prescribed and/or dispensed more than required (65, 21.2%), and self-discontinuation as the illness symptoms improved (56, 18.3%). The fridge was the most reported site for drug storage (271, 88.6%), followed by the bedroom (82, 26.8%) and kitchen (48, 15.7%). A total of 214 (69.9%) of the parents who store medication keep it at a level above adults' eyes and 58 (19%) keep it in a box, a bag, or an unlocked drawer via a key or PIN, but 28 (9.2%) keep it at a level below adults' eyes.

**Table 3:** Study parents' practice of drug storage and disposal in the Eastern region of Saudi Arabia (n = 353)

Drug storage and disposal practice	Number	Percentage %
Do you store medications at your house?		
Yes	306	86.7%
No	47	13.3%
Approximately how many medications do you store at your house		
1-5 medications	151	49.3%
6-10 medications	63	20.6%
More than 10	60	19.6%
Not sure	32	10.5%
Reason for storing medications at house		
Possible future use	205	67.0%
For daily use	187	61.1%
Prescribed and/or dispensed more than required	65	21.2%
Self-discontinuation as the illness symptoms improved	56	18.3%
Forgetfulness	44	14.4%
Change or discontinuation by the doctor	39	12.7%
Self-discontinuation due to unpleasant side effects	23	7.5%
Clear instructions not given by the doctor or the pharmacist	13	4.2%
Reached the expiry date	8	2.6%
Where do you store your medications exactly?		
Fridge	271	88.6%
Bedroom	82	26.8%
Kitchen	48	15.7%
Living room	22	7.2%
Others	8	2.6%
Storage method		
At a level above adults' eyes	214	69.9%
Box or a bag or an unlocked drawer via a key or PIN	58	19.0%
At a level below adults' eyes	28	9.2%
Others	6	2.0%

**Figure 1:**
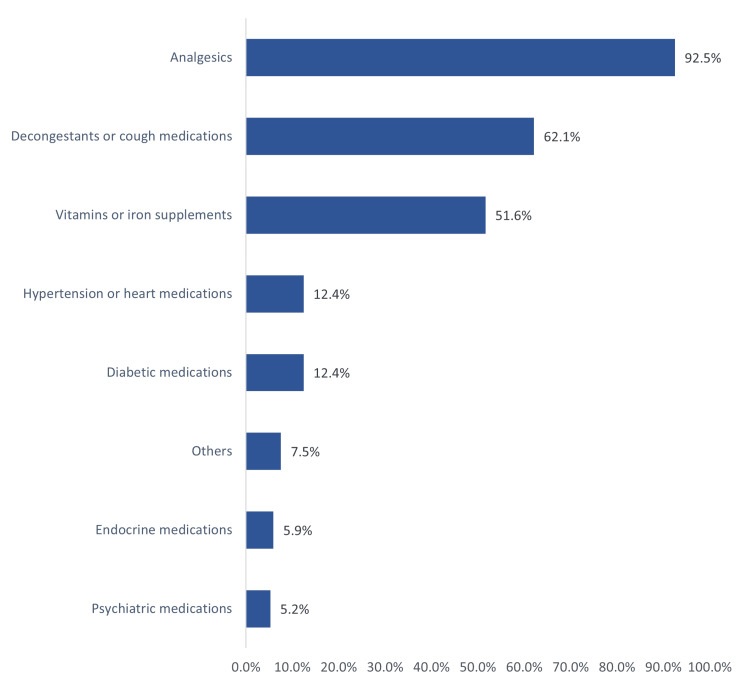
Stored medications category as reported by the study parents in the Eastern region, Saudi Arabia

The most frequently stored medications included analgesics (92.5%), antihistamine medications (62.1%), vitamin and iron supplements (51.6%), and other chronic health disease medications (Figure 1).

Thirty (96.8%) of young mothers store medications compared to 54 (79.4%) of mothers more than 45 years old with a recorded statistical significance (P = 0.046) (Table 4). Moreover, 62 (92.9%) of highly educated mothers store medications at home versus six (66.7%) of those with low education levels (P = 0.048). None of the other factors were significantly associated with medication storage.

**Table 4:** Factors associated with parents' storage and disposal of medications P: Pearson X^2^ test; ^: exact probability test; * P < 0.05 (considered significant)

Factors	Do you store medications at your house?				p-value
	Yes		No		
	Number	Percentage %	Number	Percentage %	
Age in years					0.046*
18-24	30	96.8%	1	3.2%	
25-34	113	89.0%	14	11.0%	
35-44	109	85.8%	18	14.2%	
>45	54	79.4%	14	20.6%	
Gender					0.842
Male	132	86.3%	21	13.7%	
Female	174	87.0%	26	13.0%	
Educational level					0.048*^
Below high school	6	66.7%	3	33.3%	
High school	72	82.8%	15	17.2%	
University graduate	202	88.2%	27	11.8%	
Post-graduate	26	92.9%	2	7.1%	
Family size					0.931
<4	76	87.4%	11	12.6%	
4-8	214	86.3%	34	13.7%	
>8	16	88.9%	2	11.1%	
Does anyone in your house suffer from chronic illnesses?					0.188
Yes	108	90.0%	12	10.0%	
No	198	85.0%	35	15.0%	
Have you ever received any advice regarding safe storage of medications?					0.744
Yes	138	87.3%	20	12.7%	
No	168	86.2%	27	13.8%	

Only parents' education was associated with their receiving advice about drug storage, where seven (77.8%) of low-educated mothers received advice regarding safe storage of medications compared to 11 (39.3%) of highly educated mothers (P = 0.049) (Table 5).

**Table 5:** Factors associated with parents' awareness regarding and disposal of medications P: Pearson X^2^ test; ^: exact probability test; * P < 0.05 (considered significant)

Factors	Have you ever received any advice regarding safe storage of medications?				p-value
	Yes		No		
	Number	Percentage %	Number	Percentage %	
Age in years					0.191
18-24	14	45.2%	17	54.8%	
25-34	49	38.6%	78	61.4%	
35-44	66	52.0%	61	48.0%	
>45	29	42.6%	39	57.4%	
Gender					0.452
Male	65	42.5%	88	57.5%	
Female	93	46.5%	107	53.5%	
Educational level					0.049*^
Below high school	7	77.8%	2	22.2%	
High school	32	36.8%	55	63.2%	
University graduate	108	47.2%	121	52.8%	
Post-graduate	11	39.3%	17	60.7%	
Family size					0.586
<4	35	40.2%	52	59.8%	
4-8	114	46.0%	134	54.0%	
>8	9	50.0%	9	50.0%	
Does anyone in your house suffer from chronic illnesses?					0.457
Yes	57	47.5%	63	52.5%	
No	101	43.3%	132	56.7%	

## Discussion

Across the world, people can purchase prescription or over-the-counter medications to treat either acute or chronic illnesses [17,18]. The usage of several medications contributes to dangerous medication storage practices, which result in mishandled or incorrectly stored pharmaceuticals [7,19]. The Good Distribution Practice Guidelines state that at every point in the medical supply chain, drug storage should be closely inspected and strictly regulated [20]. Unfortunately, there is a lack of proper understanding regarding the safe and appropriate storage of pharmaceuticals in many nations, which leads to the usage of medications in a hazardous manner [21].

The current study aimed to assess drug storage and disposal practices and attitudes among parents in the Eastern region of Saudi Arabia. The study results showed that most of the respondent parents were mothers at their middle age and highly educated, and one-third of them had a family member with a chronic health problem. Regarding medication storage and disposal, the vast majority of the study parents (more than three-fourths) stored medications at the house including mostly one to two drugs. Possible future use and medications for daily use were the most reported reasons for storage medications, and analgesics, cold medications, and vitamins were the most stored medications. Most of the mothers stored medication in the fridge and at a level above adults' eyes (at reach). These findings were concordant with Hendaus M et al.'s study [13], which found that 57.4% of parents stored medications at home and 90% of the parents kept their prescriptions in easily accessible locations. Ten percent of caregivers keep several prescriptions in one container, and the same proportion of participants do not check the labels for expiration dates. Antihypertensives topped the list of drugs most frequently kept in home storage in contrast to the current study list. In Ethiopia, Kahsay H et al. [22] documented that 52.4% of the respondents had unused medicines stored at home, with analgesics being the most common (41.5%). Around three-quarters (75.2%) and 63% of the respondents discarded unused and expired medicines in garbage bins, respectively. A lower practice was reported in India and Harar City, where 68% and 66% of the respondents, respectively, stored unused medicines at home [23,24]. Analgesics and antibiotics were the most reported stored medications in many literature study findings at different regions [22,23,25]. In Saudi Arabia, Al Ruwaili N et al. [26] found that over half of the participants (54.3%) kept their drugs above the average adult's eye level. Most medications (60.2%) were stored in refrigerators; the remaining 45.9% were stored in kitchens, 45.1% in bedrooms, 8% in living rooms, and 2% in bathrooms. Another study in the eastern region [11] revealed that of those surveyed, 91.0% kept their prescription drugs in their original containers, while just 4.5% marked the new containers with the expiration date. A mere 16.2% of respondents spoke with the pharmacist regarding storage instructions, despite 47.1% of respondents having read the medicine leaflet's storage instructions. When using medication, the majority of respondents (84.4%) check the expiration date, and 70.1% of them check the date of storage on a regular basis. Okumura et al. [18] assessed drug utilization and self-medication in rural Vietnam. The study revealed that 96 different antibiotics were kept at home. Furthermore, a study conducted in Sri Lanka showed that most medication accidental ingestions included analgesics (35.6%), followed by anticonvulsant (14.6%) and antihypertensive (7.6%) drugs [7].

With regard to parents' awareness and perceptions about medication storage, the current study showed that less than half of them received advice regarding safe storage of medications, which was mainly from leaflets attached to the medication package and pharmacists. Most of the parents reported throwing drugs in the garbage as the best method for disposal of unused medications with a very few percent knowing about returning them to a pharmacy or a hospital for disposal. As for parents' awareness about the effects of improper storage of medications, decreased efficacy of the drug and increased possibility of side effects were the most reported, but some of them think that medication storage in any method does not affect the quality or efficacy of the medication. About two-thirds said that the improper disposal of medications affects the environment and health. In concordance with the current study, the most common drug disposal practice in Saudi Arabia was discarding unused medications in the household garbage or flushing them down to the toilet [14,16,27,28]. Keeping medications for hazardous waste collection, giving them to friends, or burning any leftover medications were also reported practices. Al Ruwaili N et al. [26] documented that only 11% of the participants disposed of unwanted medications by returning them to pharmacies. Other studies revealed high awareness regarding environmental hazards of unsafe medication storage in contrast to the current study findings [22,23,29].

Limitations

This study had some limitations. The study's cross-sectional survey design limited our ability to determine causality between study variables. There was a lack of manpower, and the respondents self-administered the data, which may have introduced bias. In our study, we wanted to highlight patients' disposal practices. However, we did not quantify or identify the formulations of discarded medications, nor did we calculate the waste cost. Furthermore, because the study was limited to the Eastern region, the findings may not be representative of disposal practices in other regions of Saudi Arabia. However, the current results provide general ideas and a starting point for future well-designed studies highlighting the extent of medication disposal problems, investigating environmental effects, and quantifying disposed medications.

## Conclusions

These results give rise to questions regarding the proper storage and disposal of medications within the community. The environment is at risk because no household regularly returns unwanted medications to a pharmacy for appropriate disposal. More public education is required regarding the proper handling and storage of medications at home and the function of pharmacists in the workplace.
